# The Roles of FHL3 in Cancer

**DOI:** 10.3389/fonc.2022.887828

**Published:** 2022-05-24

**Authors:** Zhenjun Huang, Chengpeng Yu, Liqing Yu, Hongxin Shu, Xianhua Zhu

**Affiliations:** ^1^ Department of Vascular Surgery, The Second Affiliated Hospital of Nanchang University, Nanchang, China; ^2^ Second Clinical Medical College, Nanchang University, Nanchang, China; ^3^ Hepatic Surgery Center, Tongji Hospital, Tongji Medical College, Huazhong University of Science and Technology, Wuhan, China

**Keywords:** LIM-only protein family, four and a half LIM protein 3 (FHL3), tumor cell growth, tumor progression, cancer treatment

## Abstract

The four and a half LIM domain protein 3, also named the LIM-protein FHL3, belongs to the LIM-only family. Based on the special structure of LIM-only proteins, FHL3 can perform significant functions in muscle proliferation and cardiovascular diseases by regulating cell growth and signal transduction. In recent years, there has been increasing evidence of a relation between FHLs and tumor biology, since FHL3 is often overexpressed or downregulated in different cancers. On the one hand, FHL3 can function as a tumor suppressor and influence the expression of downstream genes. On the other hand, FHL3 can also play a role as an oncoprotein in some cancers to promote tumor progression *via* phosphorylation. Thus, FHL3 is proposed to have a dual effect on cancer progression, reflecting its complex roles in cancer. This review focuses on the roles of FHL3 in cancer progression and discusses the interaction of FHL3 with other proteins and transcription factors. Finally, the clinical significance of FHL3 for the treatment of cancers is discussed.

## Introduction

The four and a half LIM (FHL) protein family, also named the FHL LIM-protein family, belongs to the LIM-only protein superfamily. It is defined by the special arrangement of secondary structures in the LIM domain, which is composed of two tandemly repeated zinc fingers (1, 2). LIM-only proteins were originally derived from three homeodomain-containing transcription factors: (1) Lin-11, which promotes asymmetric cell division during vulval development in *Caenorhabditis elegans*, (2) Isl-1, which plays a significant role in the generation and progression of rat motor neurons, and (3) Mec-3, which controls the differentiation of neurons in *C. elegans*.

The FHL LIM-protein family includes FHL1, FHL2, FHL3, FHL4, and FHL5 (ACT, activator of CREM in testis) (3–5). The first member of the FHL protein family, FHL1, was initially named SLIM1 (skeletal muscle LIM protein1) which was initially found in skeletal muscle 24 years ago (1, 6). With the rapid development of protein detection technology, other complete protein sequences and related homologous proteins have gradually been found. Thus, the designation SILM1 was replaced by FHL1 (7, 8). The FHL protein family is composed of four complete LIM domains, which are separated by eight amino acid residues and a half LIM domain called a single zinc finger domain at the N-terminus (9). A complete LIM domain is a double zinc finger structure rich in cysteines that contains 55 amino acids (10–12). The consensus amino acid sequence is: CX_2_CX_16–23_HX_2_CX_2_CX_2_CX_16–21_CX_2_ (Cys/His/Asp) (X representing any amino acid) (10).

The FHL family members exert many different effects. FHLs are expressed in different tissues and organs (13). FHL4 and FHL5 (ACT) were mainly studied in mice. In view of having no human ortholog reported before, FHL4 is limited to spermatogenic cells in spermatogenic tubules (2). FHL5 (ACT) is also limited to the murine testis and expressed in a group of human tumors, including leukemia, melanoma, and squamous cell carcinoma cell lines (2, 9). However, FHL5 has significantly different effects depending on the context. Morgan et al. found that the expression of FHL5 was at a lower level in the H376 cell line of squamous cell carcinoma. However, FHL5 expression was higher in the Mel17 and THP-1 cell lines. Thus, the expression level of FHL5 in various tumors is different and it may also be a predictable factor in tumor regulation. Accordingly, FHL1 and FHL2 are widely found throughout the human body in different tissues (13). FHL1 is mainly expressed in skeletal muscle (14) while FHL2 is detected in cardiac muscle at a high level (15). In addition, there is a close link of FHL mutations with various myopathies. It has been discovered that mutations of the FHL gene can result in different muscular dystrophies. In most cases, the skeletal muscle lesions are accompanied by cardiovascular diseases. Moreover, the FHL family members can also function depending on the interacting protein and cell-type. It is involved in various cellular processes, including the regulation of transcription, cell differentiation, proliferation, migration, apoptosis, and signal transduction (16, 17). For example, FHL5 (ACT) can interact with cyclic adenosine monophosphate (AMP) response element modulator (CREM) to function as a transcriptional coactivator (9). Although FHL proteins can influence various processes, the regulation of transcription factor activity and the actin cytoskeleton are common functions of all FHL- proteins (18).

Four and a half LIM domain protein 3 (FHL3) is recognized as one of the least characterized proteins of the FHL family. Studies have demonstrated that the amino acid sequence of FHL3 had homology of substitution points with other FHLs. FHL3 has 40% identity with FHL1 and FHL2 but less than 20% with ACT. This indicates that FHL3 may have unique functions. FHL3 is mainly expressed in skeletal muscle and its expression pattern during *in vitro* myogenesis was found to be identical with that of FHL1 (14). FHL3 is usually located in the nucleus and contributes to the function of focal adhesions (19). It mediates protein-protein interactions and plays a significant role in transcription. In recent years, there is increasing evidence that FHL3 can also have a considerable influence on the development of cancers (20). FHL3 is overexpressed in many tumor types such as gastric cancer and glioma stem cells, while being downregulated in breast cancer. In this review, we discuss and analyze the roles of FHL3 in cancer and hope to inspire the discovery of new diagnostic methods and optimal therapies.

## The Biogenesis, Structure, and Biological Function of FHL3

**Figure 1:**
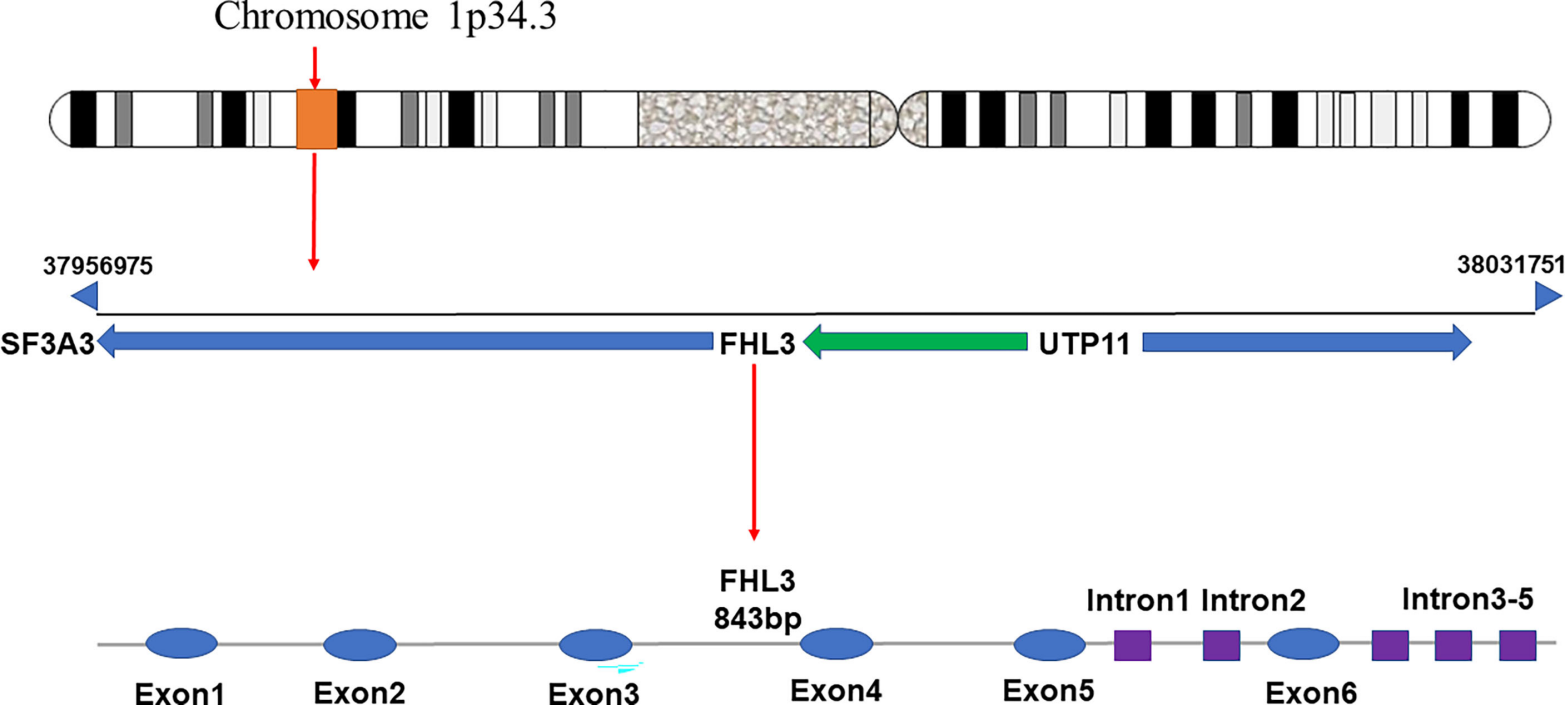
The gene encoding FHL3 is located on chromosome 1p34.3, at a locus between SF3A3 and UTP11. The initiation site is at base-pair 37956975 and the termination site is at base-pair 38031751. The gene has an overall length of 843 bp, with 5 introns and 6 exons.

**Figure 2:**
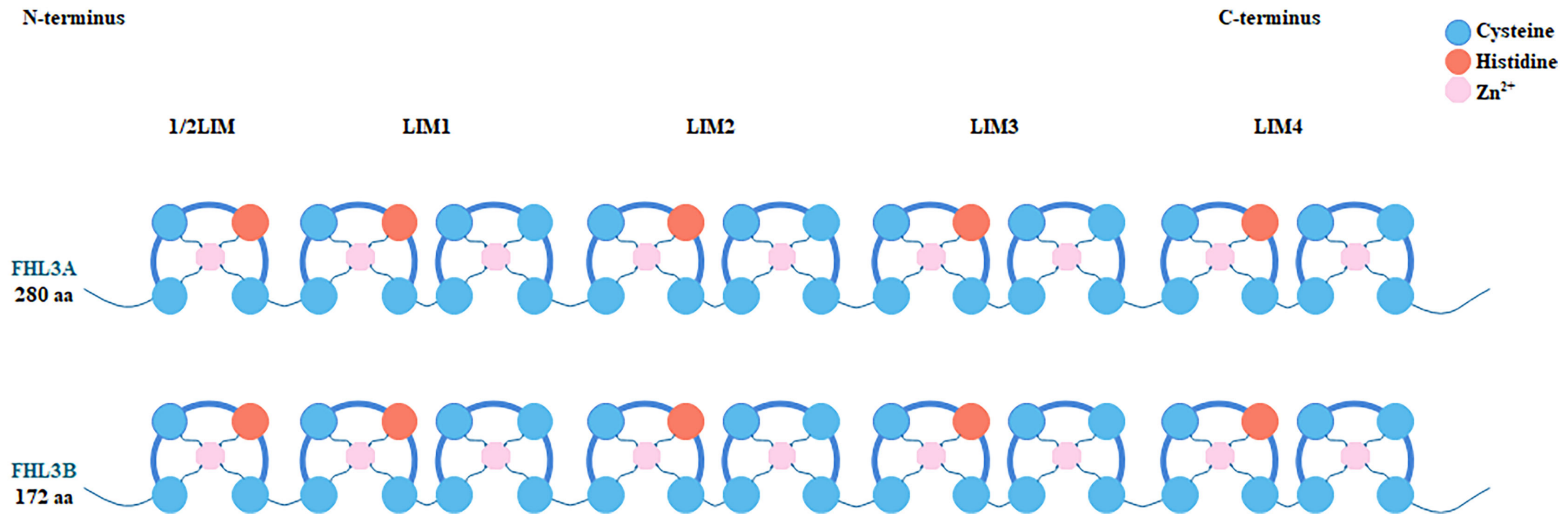
Each member of the FHL protein family consists of four complete LIM domains and an N-terminal single zinc finger domain, the sequence of the latter corresponding to the C-terminal half of the LIM motif. Histidine and cysteine residues coordinate the binding of two Zn^2+^ ions per LIM domain, which helps stabilize the secondary and tertiary structure of FHL3 protein. Two isoforms of FHL3 are shown in the figure. Isoform 1 comprises 280 and isoform 2 comprises 172 amino acid residues.

FHL3 belongs to the FHL LIM-protein family. The coding genes of this family have high sequence similarity. They are concentrated in the distal end of the short arm of chromosome 1 (21). FHL3 was first discovered based on four human heart cDNAs (22). The gene encoding FHL3 is located between SF3A3 and UTP11, at p34.3, and is about 843 bp long including 5 introns and 6 exons (**Figure 1**). Since it has 2 isoforms, the length is 1480 nt or 1686 nt. Unfortunately, the distribution of FHL3 gene expression is still unknown. FHL3 protein is composed of four full LIM-domains and an N-terminal half LIM domain. The LIM domain is an enzymatically inactive protein-protein interaction domain that determines the function as scaffold proteins or adapter molecule (**Figure 2**). Isoform1 of FHL3 is 280 amino acids (aa) in length and isoform 2 has 172 aa.

Hybridization of an FHL3 cDNA probe with poly-(A) RNA from various human tissues revealed that there is no hybridization signal in tissues other than skeletal muscle. In the mature skeletal muscle, FHL3 is localized to the Z-line. Studies have found that FHL3 is localized to the nucleus and focal adhesions in C2C12 myoblasts. After integrin binding in C2C12 myoblasts, FHL3 leaves the nucleus and breaks actin bundles by binding and cross-linking (23). Ultimately, it localizes to actin stress fibers (23). FHL3 plays a role in myogenesis and muscle reconstruction through interactions with various proteins (24–26). Due to the same nuclear location with FHL2, FHL3 can be a co-activator for the transcription factor cAMP-response-element-binding protein (CREB) and myogenic differentiation 1 (MyoD), which may represent a new mechanism for CREB-mediated transcription and MyoD-mediated transcription (27). Through this mechanism, FHL3 regulates the expression of myosin heavy chains (MyHCs) to promote differentiation of C2C12 myoblasts (26, 28). Additionally, when the expression of FHL3 increases in migrating myoblasts, the actin stress fibers will be depolymerized, suggesting that FHL3 can inhibit the actin bundle induced aggregation by α-actin (29). Moreover, when the high-affinity IgE beta chain regulator MZF-1 mediates β-chain gene transcription, FHL3 functions as a transcriptional co-repressor (25). In addition, FHL3 can also regulate cell growth and differentiation. For example, lower expression of FHL3 can suppress HIV-1 replication in HeLa-derived TZM-bl cells (21).

## The Expression of FHL3 in Cancer

There is increasing evidence that FHL3 is abnormally expressed in different cancers. FHL3 was found to be downregulated in hepatocellular carcinoma and breast cancer, indicating that it plays a role in inhibiting tumor growth. In addition, FHL3 shows high expression in gastric cancer, glioma stem cells, pancreatic ductal adenocarcinoma, non‐small cell lung cancer T-cell leukemia, A673 neuroepithelioma cells, and tumor angiogenesis. FHL3 has a tumor-promoting effect in gastric cancer and non‐small cell lung cancer. Conversely, FHL3 acts as a tumor suppressor in glioma stem cells and pancreatic ductal adenocarcinoma, which induces cell-cycle arrest. These findings indicate that FHL3 plays diverse roles in tumor progression and the details are specifically summarized in **Table 1**.

**Table 1:** The expression and roles of FHL3 in different human cancers.

Cancer type	Levels	Function	Target genes/related proteins	Ref.
Hepatocellular cancer (HC)	Down	Inhibit cell growth	p21 and c-myc	(17)
Breast cancer (BC)	Down	Suppress cell growth and induce cell-cycle stagnation at g1 and g2/m phases	p21, cyclin D1 and cyclin B1	(30)
Gastric cancer (GC)	Up	Promote metastasis and emt-mediated tumor invasion	Slug	(31)
Glioma stem cell (GSC)	Up	Suppress cell growth, proliferation, and self-renewal	SOX4 and SOX2	(32–34)
Pancreatic ductal adenocarcinoma (PDAC)	Up	Inhibit migration promote cell growth and metastasis	snail1 and twist1	(35)
Non‐small cell lung cancer (NSCLC)	Up	Promote cell growth, proliferation, and invasion	P27, TGF-β, and Smad2/3	(36)
T-cell leukemia (ATL)	Up	Increase the mobility of HTLV-1-infected and -transformed cells	TAX1	(37)
A673 neuroepithelioma cells	Up	Promote the expression of nNOS	cAMP, CREB and CREM	(38)
Tumor angiogenesis	Up	Inhibit tumor growth and progression	HIF-1	(39,40)

## FHL3 in Hepatocellular Carcinoma

Hepatocellular carcinoma (HCC) is a common malignant tumor which ranks third in the world with its high mortality rate (41). It has been confirmed that FHL3 has inhibitory effect on growth and migration of HCC (30). FHL3 was first recognized as a tumor suppressor due to its interactions with other proteins. In this way FHL3 has a positive role in inhibiting cell growth and migration. In the TGFβ/Smad signaling pathway, it has been found that transforming growth factor-β (TGFβ) activated Smad2 and Smad3 by binding to an isomer receptor complex to induce conformational changes, leading to further interactions with Smad4 and the formation of an activated complex. Ultimately, it translocated to the nucleus to regulate the expression of target genes. Thirteen years ago, Ding et al. (30) reported that FHL3 can interact with Smad2-4 independently of TGFβ. FHL3 was found to promote the phosphorylation of Smad2 and Smad3 through casein kinase 1 (CK1δ) (42), forming a complex and binding to Smad4 to translocate to the nucleus. Similar to the TGFβ-like response, FHL3 protein finally regulates the expression of tumor suppressor genes. By comparing the expression of FHL1-3 in several hepatoma cell lines (HepG2, Hep3B, and SMMC7721) and an immortalized normal human hepatocyte line (LO2), Ding et al. found that the expression of FHL3 was downregulated in liver cancer, which was correlated with TGF-β–like responses. The overexpression of FHL3 can inhibit cell growth by regulating the level of p21 and c-myc. Immunohistochemistry analysis of tissue samples from liver cancer patients and matched healthy controls verified that P21 presents decreased level and c-myc presents increased levels in HCC. In addition, knockdown of any FHL protein does not affect the levels of other FHL proteins, suggesting that there is no functional redundancy or close relation among FHL proteins. Overall, high expression of FHL3 in HCC cells may play an oncogenic role in promoting the cell growth through p21 and c-myc.

## FHL3 in Breast Cancer

The first finding that FHL3 could play a role in the progression of breast cancer came from research by Kleiber et al. (17). They demonstrated that FHL1-3 can interact with Smad2-4 and influence the development of cancer (30). FHL was found to inhibit hepatocellular carcinoma cell growth and proliferation *in vitro* and *in vivo* (30). Similarly, FHL3 was found to be downregulated in breast cancer cells compared with normal cells (17). Overexpression and silencing demonstrated that FHL3 can inhibit the growth of breast cancer cells by inducing cell cycle arrest in the G1 and G2/M phases. It is also associated with the inhibition of cyclins D1 and B1. It has been reported that overexpression of cyclin D1 in MCF7 cells leads to continued proliferation in the absence of growth factors, while non-expressing cells cease to grow (43). These data indicate the role of these factors in the development of breast cancer. Similar to cyclin D1, cyclin B1 has been shown to generally inhibit breast cancer cell proliferation (44). In addition, the knockdown of cyclin B1 is related to taxol treatment (45). At the same time, it also enhances cyclin-dependent kinase inhibitor P21 which is considered a G1 and G2 molecule (46, 47), suggesting that FHL3 plays a significant role in regulating the cell cycle. Based on these findings, it can be hypothesized that the regulation of P21 expression in breast cancer cells may be influenced by the interactions of FHL3 with Smad proteins, although it cannot be ruled out that FHL3 may affect P21 expression *via* other protein partners. The details of the underlying mechanisms remain to be elucidated.

## FHL3 in Gastric Cancer

Gastric cancer (GC) ranks fifth in mortality among all malignancies since the effect of adjuvant chemotherapy after surgical treatment is usually unsatisfactory. Cao et al. (31) found that FHL3 is a biomarker for predicting disease progression and prognosis in GC. The expression of FHL3 mRNA is upregulated in GC, and higher expression was correlated with lower differentiation, increased metastasis, and worse TNM stage (31). In addition, the knockdown of FHL3 was found to reduce tumor growth (31). The epithelial mesenchymal transition (EMT) is considered the main cause of tumor metastasis, and the down regulation of e-cadherin is a major characteristic of the EMT process (48, 49). FHL3-induced EMT was correlated with the activation of the MAPK/ERK/JNK/P38 and PI3K/Akt/GSK3β pathways. Conversely, the downregulation of FHL3 can promote the expression of E-cadherin and reduce the migration ability of tumor cells. Furthermore, TGFβ/Smad-independent pathways are also involved in FHL3-mediated chemotherapy resistance. Slug, which can suppress the activity of caspase 9, increases the chemoresistance of tumor cells (50). FHL3 can upregulate Slug expression by competitively binding ubiquitin in complex with Slug, which leads to the metastasis of GC. Overall, FHL3 appears to regulate the progression of GC through various pathways, promoting the EMT and chemotherapy resistance.

## FHL3 in Glioma Stem Cell

Glioma is the most common malignant primary brain tumor in adults, with a high morbidity and mortality worldwide. It is characterized by rapid metastasis, aggressive infiltration, and poor prognosis (51–53). According to the WHO 2016 criteria, the histological classification of gliomas is defined from grade I to IV (32). Grade IV glioblastoma multiforme (GBM) is an overly aggressive tumor that accounts for about three-quarters of all gliomas (54). Xia et al. revealed that angiogenin (Ang) may regulate the expression of FHL3 and further activate the NF-κB pathway (55). High FHL3 levels were found to inhibit U87MG cells, while FHL3-knockdown increased the phosphorylation of IκBα and the overexpression of Ang. Another study found that FHL3 is required for nuclear translocation of Ang and Ang-mediated HeLa cell proliferation (56). It highlights that FHL3 plays a role in the molecular mechanisms driving the apoptosis resistance of cancer cells. In addition, FHL proteins also regulate the proliferation and differentiation of various cells through interactions with other proteins. Inhibition of PCBP2 enhanced FHL3 expression by stabilizing its mRNA, leading to the increased expression of P21 in glioma cells (33). Furthermore, the study also showed that it can increase the expression of P16 and decrease the expression of P27 in FHL3-induced T98G cells. Notably, subsequent studies revealed that the proliferation of non-stem glioma cells is prevented by the overexpression of FHL3 (34). In glioma stem cells, FHL3 inhibits the Smad2/3-SOX4-SOX2 axis. SOX4 has a dual role in regulating tumor behaviors. In the past, it has been indicated that SOX4 has a suppressive effect on the cell growth of glioma stem cells (57). However, it was confirmed that SOX4 can also promote tumor progression by interacting with the transcription factors Smad2/3 as well as dephosphorylating PPM1A and FHL3, which decreases the self-renewal capacity of glioma stem cells (34).

## FHL3 in Pancreatic Ductal Adenocarcinoma

As a highly malignant tumor that accounts for 90% of pancreatic cancers, the incidence of pancreatic ductal adenocarcinoma (PDAC) is on the rise (58, 59). Stage I/II tumors are curable by excision, but therapeutic options are minimal for patients with recurrent or unresectable tumors (60). The EMT is closely related to many EMT-associated transcription factors (EMT-TFs), leading to the loss of apical-basal polarity in epithelial cells and ultimately resulting in tumor metastasis (35, 61). There is evidence of the promotion of EMT by FHL3 through the TGFβ/Akt/GSK3β/ubiquitin pathway (35). FHL3 promotes the expression of Akt by enhancing the transcriptional level of TGFβ. GSK3β is the target gene of Akt, which regulates the degradation of snail1 and twist1. When FHL3 expression was downregulated, the activity of Akt was also weakened. Then, GSKβ will accordingly be activated, leading to the upregulation of snail1 and twist1. In addition, it was also found that FHL3 can bind to GSKβ to inhibit the interaction of snail1 and twist1. Thus, FHL3 can promote the expression of snail1 and twist1 *via* the TGFβ/Akt/GSK3β/ubiquitin pathway.

## FHL3 in Non‐Small Cell Lung Cancer

Non-small cell lung cancer (NSCLC) is the most frequent form of lung cancer, accounting for over 85% of all lung cancer cases (62). A lack of early screening and late-onset clinical symptoms results in an abysmal 5-year survival rate of NSCLC patients of only 20% (63). Hence, early diagnosis is crucial for the clinical outcomes of NSCLC. Hou et al. explored the function and mechanism of circGRHPR in NSCLC (36). The authors found an interaction between circGRHPR and PCBP2, whereby decreased PCBP levels can stimulate the expression of FHL3. The function of FHL3 in NSCLC appears to be similar to gastric cancer. It acts as a tumor oncogenic factor, which promotes lung cancer cells growth, proliferation, and invasion. Thus, FHL3 was demonstrated to be associated with poorer survival and prognosis. Furthermore, it was speculated that FHL3 can regulate the TGF-β-Smad2/3 axis and inhibit the expression of P27 to enhance tumor proliferation. These studies revealed a significant role of FHL3 in NSCLC, indicating that it may be a potential therapeutic target.

## FHL3 in Adult T-Cell Leukemia

Adult T-cell leukemia (ATL) is an aggressive drug-resistant hematological malignancy tumor secondary to HTLV-1 retrovirus infection (37). The prevalence of HTLV-1 is mainly attributed to vertical transmission *via* breast feeding. In addition, HTLV-1 can also be transmitted through blood transfusion, needle sharing, and sexual contact (64). The effect of HTLV-1 on T cell transformation and subsequent development of ATL is closely related to the viral regulatory protein Tax (65). McCabe et al. identified a functional interaction between Tax1 and FHL family proteins (65). As indicated by co-immunoprecipitation assays and direct protein binding studies in mammalian cells, Tax1-FHL interaction leads to their redistribution in cells. By increasing the cell-wide dispersion of FHL3, TAX1 also affects the cytoskeletonregulator function of FHL3 (66). On the other hand, Tax1-mediated activation of viral long terminal repeat (LTR) and NF-κB pathways is enhanced by FHL3 in T-cells. The specific mechanism of action is in line with a recent study, which found that Ang activates the NF-κB pathway by regulating the expression of FHL3 (55). Notably, the effect of FHL3 on the activation of the NF-κB pathway mediated by Tax1 varies according to the type of affected cell. Thus, Tax1 has a significant role in promoting the expression of FHL3 to enhance its wide dispersion, demonstrating that Tax1 can interact with FHL3 to increase the mobility of HTLV-1-infected and -transformed cells.

## FHL3 in A673 Neuroepithelioma Cells

Neuroepithelioma is a rare invasive tumor that affects young adults (67). In the A673 neuroepithelioma cell line, cAMP analogues and compounds that increase the intracellular levels of cAMP were found to promote the expression of human neuronal nitric oxide synthase (nNOS) by promoting the activity of mRNA/protein (38). The nNOS enzyme generates NO, which is involved in various biological functions such as neuroprotection (68). In addition, nNOS can also produce reactive oxygen species (ROS), which can contribute to different pathological changes such as Alzheimer’s disease (69, 70). Furthermore, cAMP response element binding and modulator transcription factors (CREB and CREM), which interact with PKA, can enhance the synergy between PKA and nNOS. It was also found that FHL3 is expressed in A673 neuroepithelioma cells, where it activates CREB and CREM, thereby upregulating the PKA-independent pathway. Thus, FHL3 may play a key role in the expression of nNOS in A673 neuroepithelioma cells.

## The Interaction Between FHL3 and Hypoxia-Inducible Factor 1 (HIF-1)

Hypoxia-inducible factor 1 (HIF-1), which consists of α and β subunits, is an important transcription factor in the process of angiogenesis and other critical aspects of cancer biology (71). HIF-1 is commonly overexpressed in different cancers (72), where it can regulate the expression of target genes to harmonize the adaptive response to tumor hypoxia (73). In the past, it was found that FHL3 can inhibit both HIF-1α and HIF-1β (39). Unfortunately, the underlying mechanism remains unknown. Recent studies have found that FHL3 can decrease the expression and promoter activity of vascular endothelial growth factor (VEGF) in tumor angiogenesis by inhibiting both HIF-1α and HIF-1β (40). FHL3 can interact with FHL1-2 to suppress the expression of VEGF mRNA and protein, leading to the degradation of HIF1 and inhibiting tumor angiogenesis.

## Conclusions and Perspectives

**Figure 3:**
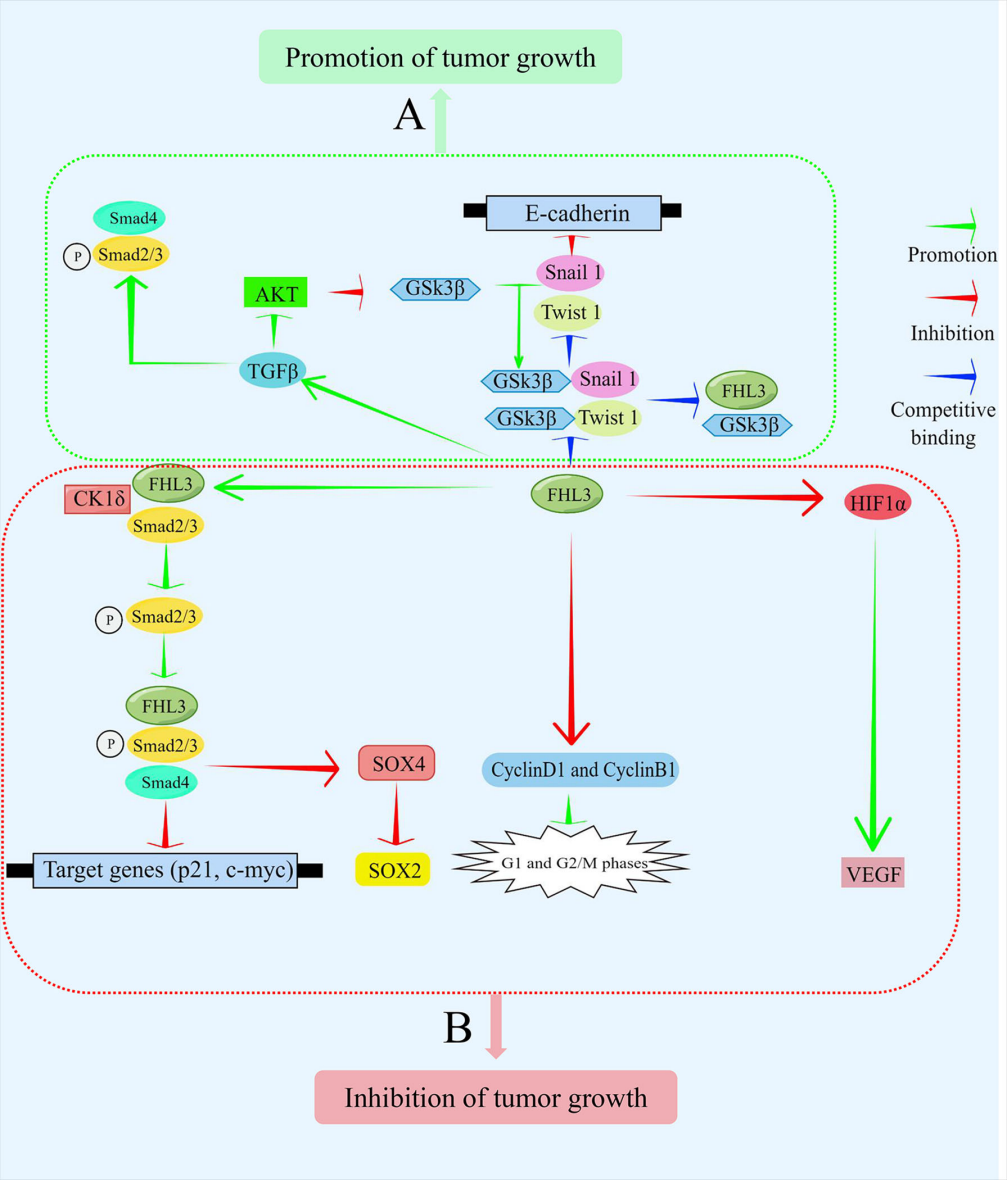
FHL3 functions as an oncoprotein or tumor suppressor depending on the cellular context. It mediates the transduction of signaling pathways by interacting with other proteins, thereby controlling the expression of target genes. **(A)** In some contexts, FHL3 functions as a tumor promoter. FHL3 competitively binds GSK3β to interfere with Snail1 and Twist1 to inhibit the expression of its target gene, E-cadherin. Additionally, FHL3 activates Smad2, Smad3, and Smad4 *via* TGFβ to promote tumor growth. **(B)** In other contexts, FHL3 functions as a tumor suppressor. FHL3 promotes the phosphorylation and binding of Smad2 and Smad3 with CK1δ. Subsequently, the expression of the target genes, p21 and c-myc, can be decreased to inhibit tumor growth. SOX4 and SOX2 are also target genes that are downregulated to inhibit tumor growth. Additionally, FHL3 can inhibit cyclinsD1 and B1, affecting the G1 and G2 checkpoints. Moreover, FHL3 inhibits the expression and activity of VEGF by inhibiting HIF1α.

FHL3 belongs to the LIM-only protein family, which is recognized as one of the least characterized in the FHL superfamily. The protein is mainly expressed in skeletal muscle and plays a significant role in diverse cellular activities. It can mediate protein-protein interactions and influence different signaling pathways such as TGFβ/SMAD, AKT, and GSK3β. Furthermore, it has also been found that FHL3 can induce the phosphorylation and ubiquitination of downstream proteins (**Figure 3**). Due to the diverse and complex roles of FHL3 in cancer progression, more studies on the detailed mechanisms are needed.

FHL3 plays crucial roles in different cancers including hepatocellular carcinoma, breast cancer, gastric cancer, glioma, pancreatic ductal adenocarcinoma, non‐small cell lung cancer, T-cell leukemia, A673 neuroepithelioma cells, and tumor angiogenesis. Early studies found that FHL3 was widely downregulated in cancers and could inhibit tumor growth. However, there is increasing evidence that FHL3 is upregulated in other malignancies, indicating that it plays dual roles depending on the context. FHL3 can suppress tumor growth in glioma stem cells and pancreatic ductal adenocarcinoma, but it can also promote metastasis in gastric cancer. Thus, FHL3 plays diverse roles in tumor progression, acting as either a tumor suppressor or oncoprotein. According to existing studies, FHL3 is more likely to act as a tumor suppressor. A more detailed understanding of the roles of FHL3 as a tumor suppressor or oncoprotein will have profound implications for cancer treatment. FHL3 may be a potential therapeutic target in different cancers, with potential for new prevention and treatment strategies.

## Author Contributions

HS and LY collected the related paper. ZH drafted and revised the manuscript. CY designed the review. XZ participated in the design of the review and helped to draft and revise the manuscript. All authors read and approved the final manuscript.

## Conflict of Interest

The authors declare that the research was conducted in the absence of any commercial or financial relationships that could be construed as a potential conflict of interest. 

## Publisher’s Note

All claims expressed in this article are solely those of the authors and do not necessarily represent those of their affiliated organizations, or those of the publisher, the editors and the reviewers. Any product that may be evaluated in this article, or claim that may be made by its manufacturer, is not guaranteed or endorsed by the publisher.
